# Effect of the Sous-Vide Method on the Quality of Vegetables—A Review

**DOI:** 10.3390/foods15020206

**Published:** 2026-01-07

**Authors:** Artur Głuchowski, Ewa Czarniecka-Skubina, Marlena Pielak

**Affiliations:** Department of Food Gastronomy and Food Hygiene, Institute of Human Nutrition Sciences, Warsaw University of Life Sciences (SGGW-WULS), 02-787 Warsaw, Poland; artur_gluchowski@sggw.edu.pl (A.G.); marlena_pielak@sggw.edu.pl (M.P.)

**Keywords:** sous-vide, cooking method, vegetables, quality, nutritional value, sensory quality, microbiological quality

## Abstract

Modern gastronomy strives to combine high-quality food with the preservation of nutritional value, microbiological safety, and the sustainable use of raw materials. With the development of culinary technologies, precise heat treatment methods are gaining increasing importance, enabling better process control and more consistent quality results. This analysis aims to present the effects of the sous-vide (SV) method on the quality of vegetables in comparison with conventional heat treatment methods, such as boiling in water, steaming, cooking under increased pressure, cooking in a microwave oven, baking, grilling, and the cook-vide method. Analysis of the scientific literature has shown that the sous-vide method usually allows for the retention of greater amounts of vitamins (especially vitamin C), phenolic compounds and minerals, resulting in products with higher nutritional value and bioavailability of bioactive ingredients. Maintaining a controlled, low temperature in a vacuum environment reduces the loss of water and volatile components, which has a positive impact on the process yield as well as the color, texture, and aroma of vegetables. SV processing enhances product digestibility, preserves natural appearance, and improves food safety. Due to its hermetic packaging and limited oxygen access, this method ensures good microbiological quality and extends product shelf life. In the food service industry, SV allows for repeatable results, high sensory and technological quality, and reduced food waste. In the context of contemporary nutritional challenges and the experiences of the COVID-19 pandemic, sous-vide technology is gaining importance as a method supporting food safety, sustainability, and efficient resource management in the food service industry.

## 1. Introduction

Sous-vide (SV) is a method of heat-treating food at a precisely controlled temperature, usually lower than conventional temperatures, after placing it in an airtight, usually vacuum-sealed package. The goal is to achieve high-quality, nutritional value, and extended shelf life. Low-temperature heat treatment using the SV method involves 5 stages: pre-treatment, including the use spices; pre-heat treatment to impart characteristic flavor and aroma (Maillard reactions); vacuum packaging or modified atmosphere packaging; pasteurization under controlled temperature and time conditions in a water bath or in a convection-steam oven [1,2]; and expedition—SV products can be served immediately after heat treatment or cooled in the packaging to a specified temperature (0–3 °C), stored in a cold store and pasteurized immediately before serving [3].

The SV method was initially popular as a storage method and gained recognition in the meat industry for the preparation of ready-made meals; hence, many researchers focused on meat research [2,4,5,6,7,8,9,10,11,12,13]. After 2010, the SV method began to be used in other food production sectors. In recent years, SV has gained widespread use in the catering industry as a cooking technique for animal and plant-based ingredients—not only in fine-dining restaurants but also in catering companies, the convenience food industry, and the retail sector (supermarkets, fast food), where repeatability, extended shelf life, and the efficient use of resources are valued.

The sous-vide (SV) cooking technique, renowned for its precision and ability to extend food’s shelf life, has become a revolutionary method in the culinary arts [14]. The results indicate that while SV offers benefits in terms of consistent results and extended shelf life, challenges remain, particularly those related to equipment costs and the need for specialized training. While this method is sufficient for food preparation/processing, its effectiveness in eliminating microbial pathogens, including viruses, parasites, and vegetative and spore-forming bacteria, may be limited if inappropriate temperatures are used. Cooking food only at precisely defined temperatures effectively eliminates harmful pathogens, making it a safe method for preparing a variety of foods. This aspect of SV cooking is crucial for food service establishments where food safety is a priority [15].

However, research highlights SV’s adaptability and potential for culinary innovations that meet modern requirements related to food safety, quality, and nutritional value. SV enables the production of ready-to-eat foods, which reduces food waste and simplifies cold chain logistics [14,16]. The nutritional aspect of SV cooking is important because products prepared by this method effectively protect vitamins and minerals that are often lost in traditional cooking methods. This is particularly important for health-conscious consumers. The vacuum packaging process in SV cooking also inhibits bacterial growth, thus extending the shelf life of food products. This is crucial for reducing food waste and increasing food safety in global distribution and storage [14,17].

This method has gained great popularity in restaurants and other foodservice establishments, largely due to the possibility of precise temperature control and, consequently, consistent sensory quality. Precise temperature control may increase the tenderness and juiciness of meat and seafood, as well as improve the bioavailability of nutrients in vegetables. Sous-vide can also be used in households, but the full potential of the technique is often realized in professional settings where high hygiene and technological standards are maintained, and equipment and knowledge are more readily available than in a home environment without professional resources, and where the final results can be limited. Furthermore, due to its significant energy consumption (*p* < 0.001), which is 5–10 times higher than conventional methods such as boiling and steaming, it is less practical for home use. Nevertheless, the method’s effectiveness can be appreciated in both environments [14,18,19,20,21,22,23,24]. Despite its numerous benefits, SV technology also presents challenges, including the risk of insufficient microbial inactivation at too low process temperatures, the need for specialized equipment, potentially higher energy consumption, and the safety issues of storing products cooked for long periods under anaerobic conditions. Considering these issues allows for a more comprehensive assessment of the potential and limitations of this method.

Sous-vide cooking is constantly evolving; it symbolizes culinary innovation, combining tradition with modern technology. Its impact on food safety, nutritional preservation, and culinary creativity makes it an important step forward in contemporary gastronomy and food science. With its emphasis on precision, flavor enhancement, and nutritional preservation, SV cooking is redefining culinary arts, offering new opportunities to create high-quality, nutritious, and flavorful dishes in gastronomy [17,25]. However, in the food service applications, this method has its limitations. It requires a high level of hygiene, temperature control, and appropriate staff training. Furthermore, extended processing times and high energy consumption may constitute economic constraints, especially in smaller foodservice establishments or households.

The SV technique has been studied for a wide array of many vegetables, including courgette [26], potatoes [26,27,28,29,30,31,32,33], asparagus [1,32], carrots [26,28,32,33,34,35,36,37,38,39,40,41,42,43], beetroots [32,44], root parsley [45], celeriac [31,46], green peas [29,32,39,40,44,47,48,49], beans [28,32,44,48,49], red lentils [48], broccoli [26,39,42,44,47,50,51,52,53,54], *Brussels sprouts* [34,38,55,56,57,58,59,60], cauliflower [32,52,53,59,60,61], pumpkin [62,63], artichoke [39,64], purple eggplant and zucchini [31,32], spinach [50], kale [52,53,65,66]; white cabbage [52,53], red cabbage [52,53,67], fennel, squash, onions, parsnips, turnips, celery roots [32], and chickory [68].

Despite the various results and literature reviews on SV technology, the dynamic development of this method in the last decade, especially in the context of vegetables, the importance of which is growing both in diet and in the catering industry, justifies the need to update it. The new data concern not only qualitative aspects but also microbiological and sensory aspects, as well as the impact of physicochemical processes occurring during processing, which makes this review an important supplement to previous studies. The selection of different vegetables for preparing meals is driven by variations in their texture, color, and aroma, which directly impact changes in sensory quality and nutritional value, and indirectly influence microbiological safety.

In this review, we present information on SV as a method of vegetable cooking, including its impact on vegetable quality and the relationship between vegetable quality and its physicochemical and sensory indicators, nutritional value, bioavailability, digestibility, as well as microbiological quality. The paper aims to present the mechanisms of changes occurring in vegetables responsible for the desired features of products, with particular emphasis on health and technological benefits, as well as the possibilities of applications in the catering industry. In this review, the concept of “mechanisms of change” refers to physical (e.g., texture modification), chemical (e.g., degradation of bioactive components, browning reactions), and microbiological (e.g., reduction in microbial populations) factors that determine the final quality of vegetables prepared by the SV method.

Hence, to fill the scientific gaps in this area, the aims of this paper were as follows:

(1) to identify and understand the differences between SV cooking parameters and vegetable quality, as well as compare with other cooking methods,
(2) to identify and understand the preferences and opinions of consumers regarding SV technology,
(3) to identify the strengths and weaknesses, opportunities and barriers to implementing this vegetable cooking method in the food service industry, using a SWOT analysis as a strategic tool.

## 2. Materials and Methods

All data presented in this review were summarized from the references of scientific journals. These references were systematically searched in the following databases: Web of Science, Scopus, and Google Scholar. In each database, the following search string was used, adapted to the syntax of the database: (“sous-vide”) AND (“vegetables” OR “plant-based foods”) AND (“quality” OR “nutritional value” OR “sensory quality” OR “microbiological safety” OR “consumer acceptance”). First, the search term (“sous-vide”) AND (“vegetables” OR “plant-based foods”) was entered. To obtain specific results, additional terms were added, i.e., (“sous-vide”) AND (“vegetables” OR “plant-based foods”) AND (“quality” OR “nutritional value” OR “sensory quality” OR “microbiological safety” OR “consumer acceptance”). The search was conducted in the title, abstract, and keyword fields. In the Web of Science and Scopus databases, results were limited to peer-reviewed articles and books, while in Google Scholar, only publications from scientific journals were analyzed.

To search for maximum relative references, publication years were limited to 2010–2025. To explain some of the processes, references were made to earlier sources.

The criteria for including articles in the review were as follows:

1. Title and keywords: sous-vide, vegetables, quality, nutritional value, sensory quality, microbiological value, and consumer acceptance;
2. English language,
3. Source category: peer-reviewed articles and books, indexed in databases;
4. Publication period: 2010–2025.

The exclusion criteria for the review were as follows:

1. Other languages than English,
2. Non-peer-reviewed source.
3. References before 2010.
4. Works not related to sous-vide technology, research concerning only meat and fish products.

We identified records up to 2 November 2025, and found 172. Due to some records being duplicated or off-topic, 25 records were rejected. Then we identified 147 references. A two-stage publication selection process was then carried out. In the first stage, titles and abstracts were assessed for their relevance to the review topic, and in the second stage, full-text publications were analyzed. Two independent authors selected the records, and in case of discrepancies, decisions were made by consensus. Ultimately, 95 publications comparing SV technology with other heat treatment methods were included in the review.

## 3. Sous-Vide Processing Parameters

The scope, process conditions, and equipment sophistication vary depending on the setting, from basic home kitchens to industrial food production. Restaurants typically use precise temperature-controlled water baths and high-quality vacuum sealers, which ensure uniform cooking results and meaningfully improve the sensory qualities of food [3]. It is interesting how this technique affects the quality of plant raw materials, which have a different cellular structure than meat.

Analysis of recent reports confirms that one of the limitations of using SV at home is the time and energy required to heat the water bath—this is particularly noticeable for small batches. In the food service industry (production facilities, cook-chill lines), this cost is offset by efficiency gains, raw material savings, and extended shelf life. In practice, a wide range of parameters is used for vegetables (e.g., 60–100 °C; processing times ranging from several minutes to several dozen hours in the case of long-term, low-temperature processes), Table 1 [44,48].

The SV method typically recommends cooking vegetables above 80 °C because at this temperature, cell walls remain intact, the structure (fiber and pectin) softens, enzymes are inactivated, and pathogens are destroyed. However, for starchy vegetables, a slightly lower temperature, below 80 °C, is used to avoid texture changes caused by gelatinization [1,62,69].

However, using low temperatures and long cooking times (e.g., 68 °C for 24 h in the cabbage study) results in softness and shorter chewing time, which may be important for people suffering from dysphagia. The SV method softens vegetables while maintaining their structure and juiciness. Combined with high-pressure cooking, SV may be a recommended method for preparing vegetables for the geriatric population, especially in the context of easily digestible cuisine [31,46,70].

Sous-vide cooking times can vary significantly depending on the type of vegetable and the temperature used. For example, broccoli and beetroot were tested at 85 °C for 30, 60, and 180 min, which showed that longer times affected the color and texture of the vegetables negatively [44]. The use of shorter SV processing times also positively influenced the preservation of mineral components [44,48]. Due to the impact on bioavailability and metabolism, the authors consider the SV method at shorter times and higher temperatures as an alternative to steaming [55,56,64].

The choice of parameters depends on the goal we want to achieve, e.g., texture optimization, enzyme inactivation, color preservation, or nutrient retention. It should be emphasized that differences in results depend on three main sources of variability:

(1) different geometry and thickness (affecting the heating kinetics),
(2) applied process parameters (temperature, time, presence of water in the packaging),
(3) the condition of the raw material (variety, maturity, storage conditions). Careful adjustment of parameters (e.g., shortening the application time at high temperature to preserve the color) is crucial to achieving the desired results.

## 4. The Effect of the Sous-Vide Method on Yield and Water Content in Vegetables

Most studies [29,57,71] indicate a higher water content in SV vegetables compared to those prepared using other thermal processing methods (boiling in water, steaming). The authors attribute better water retention in the vegetables to the use of sealed bags, which limits water evaporation from the product. Differences between individual studies may result from different process conditions, such as time, temperature, the use of water in the bag, or cooking without water. The effect of SV cooking on the water content of the product also resulted in differences in process yield. The yield of the SV method is higher at lower process parameters than the microwave method, but lower than boiling in a pot and steaming. Vacuum sealing in the SV technique reduced moisture loss by evaporation during cooking [1,68].

Higher yields were obtained for asparagus cooked SV at 80 °C for 15 min than for samples cooked SV at 99 °C for 5 min and cooked SV in a microwave for 1.5 min. The yield of this method was lower than that of boiling and steaming, but higher than that of microwave cooking, which causes significant water evaporation (*p* < 0.001) [1]. Similar results for chicory were found by Renna et al. [68] and Muñoz et al. [29]. In the case of chicory, the yield of the SV method was statistically significantly (*p* < 0.001) lower than that of boiling in water and steaming, but higher than that of microwave cooking [68].

For plant-based products with a high protein content or a meat-like texture (e.g., textured soy protein), the SV method can increase moisture and improve the rheological properties of the structure (soft, juicy texture).

Furthermore, the potential of SV processing was explored as a technique for transforming textured soy protein (TSP) into a product with a texture comparable to meat. During the process, the soy protein maintained a moisture content of approximately 70%. Its porous microstructure changed, and with process time, the hardness and texturization index of TSP decreased. Furthermore, the protein secondary structure shows an increase in β-pleated and α-helical structures, resulting in an increased number of hydrogen bonds, hydrophobic interactions, and disulfide bonds. A soy sample processed for 24 h at 90 °C exhibited textural characteristics similar to those of chicken breast. This study indicates that the SV method is a revolutionary technique that allows for high moisture content, resulting in better texture TSP [72].

## 5. The Effect of the Sous-Vide Method on Vegetable Texture

Research on vegetables prepared using the SV method focuses mainly on the influence of specific parameters on hardness, as well as low process parameters and vacuum packaging conditions on color. Data on the taste and aroma of vegetables processed by the SV method are limited. Plant-based products require higher heat treatment temperatures than animal or fish products. Achieving the desired texture, which is related to the tissue softening process, is a key factor in determining quality. The texture of plant-based products depends on the type of cells that make up the tissue, their organization, the amount of air in the intercellular spaces, turgor pressure induced by osmosis, and the composition of the cell walls. Most edible parts of fruits and vegetables are composed of parenchyma cells. However, some raw materials, such as asparagus, are composed of vascular cells and sclerenchyma, typical of immature stems. In turn, the texture of starchy vegetables is also influenced by the starch gelatinization process [73].

The primary cell wall consists of a cellulose-hemicellulose network, in which pectin polymers are located. Polysaccharides play a crucial role in determining the structural integrity of plant cells. Thermal processing disrupts the semipermeable nature of the cell membrane, allowing soluble substances and water to diffuse freely between cells, resulting in a loss of cell turgor pressure. Changes also occur in the structure of the cell wall polysaccharides and the entire cell (primarily in pectin). The β-elimination reaction is mainly responsible for the degradation of pectin, which involves the breakdown of glycosidic bonds in pectin (in a non-acidic environment). Hydroxide ions catalyze this reaction, which depends on the temperature, pH, and degree of pectin esterification. The softening process is aided by the breakdown of the cell wall and the reduction in the middle lamella, which facilitates cell separation. In raw materials with large intercellular spaces, the movement of air released from intercellular spaces, together with cell juices, can further affect their texture [73].

For this reason, Baldwin [3] recommends cooking non-starchy vegetables (e.g., carrots) at 82–85 °C for three times longer, and starchy vegetables (e.g., potatoes) at 80 °C for twice the time needed for cooking in boiling water. He recommends cooking legumes at 90 °C for 3–6 h. Based on the literature review, it was found that a wider temperature range of 80–117 °C was used for SV potatoes (including the high-temperature sous-vide—HTSV method), 75–85 °C for carrots, and 60–100 °C for broccoli. The use of temperatures of 80–90 °C for plant products may cause the packaging to balloon, which is caused by the expansion of residual air created after vacuum packaging (remaining in porous raw materials) and the conversion of water into steam. The hardness of vegetables cooked SV decreases with increasing cooking time and temperature, with temperature playing a more important role. It should be noted that the SV method allows for a more uniform hardness throughout the vegetables [27,28,40,49], which depends on the dimensions of the raw material input [34].

The study by Guillén et al. [39] suggests that the hardness of vegetables cooked using the SV method does not differ significantly (*p* < 0.001) from that of vegetables prepared by conventional cooking methods. According to other authors [28,49,51], products subjected to limited or no direct contact with water during thermal processing (SV, microwave cooking, grilling) have less damaged tissue structure and are harder.

Potatoes are a raw material commonly used as a starch additive. Therefore, studies of potatoes processed using the SV method focused primarily on their hardness and starch transformation. Potatoes cooked SV at 90 °C (25–35 min) had a hardness similar to that cooked in a vacuum pot at 90 °C (25–35 min) or boiled at 100 °C (20–30 min). Increasing the process temperature to over 100 °C resulted in a softer product (lower hardness) [29], while lowering the temperature to 80 °C led to a harder texture compared to conventionally cooked potatoes. This can be explained by the fact that the β-elimination reaction of pectin, the main component of the potato middle lamella, increases at this temperature [28].

Iborra-Bernad et al. [27] determined that vacuum packaging, due to the separation of the aqueous medium from its contents, results in increased adhesiveness of potatoes.

The authors suggest that this is the result of the increased viscosity of sugars released from damaged cells. In an aqueous environment (traditional and cook-vide methods), however, sugars are washed out, resulting in lower adhesiveness [27]. In raw potatoes, starches are in their native form (isodiametric polyhedral cells). During thermal processing with water, they gelatinize; starch absorbs available water and generates its swelling pressure. This phenomenon was more pronounced in samples cooked conventionally and by the cook-vide method, due to the high water availability. The starch in SV potatoes uses only the water available in the cells, while those prepared as cook-vide potatoes utilize water from the cooking environment [28].

It should be noted that the starch changes in potatoes subjected to the high process parameters of 116–118 °C (HTSV method) depend on the starch content. The processing yield of high-starch varieties using the SV method is similar to that of conventional cooking; the processing yield of lower-starch varieties is comparable to that of microwave cooking. The sterilization heat doses applied in the SV method have a similar effect to traditional heat treatment methods [29].

The content and structure of starch (amylose-to-amylopectin ratio, granule size, and quality), as well as its ability to gelatinize and swell in the presence of water, largely determine the response of the potato tuber to the SV process. High-starch varieties, due to intense gelatinization, tend to have a softer, looser texture after processing. In contrast, lower-starch varieties retain a firmer and more compact structure (as evidenced by reports of “waxy” starch) [74,75,76].

Carrots. Studies [28,35] have shown that temperature has a greater effect than processing time on the softness of carrot tissue. It has been demonstrated that, unlike conventional methods, vacuum cooking methods (SV and cook-vide) result in similar hardness of both types of plant tissues (xylem and phloem). A more uniform consistency can be obtained by using longer heat treatment times. This process is characterized by different heat transfer kinetics. In the study by Tansey et al. [37], a linear decrease in the hardness of SV carrots (85 °C) was observed with increasing process time up to 50 min, after which the rate of decline slowed. The optimal processing time was set at 60 min. However, it depended on the variety, the type of heat medium (water or steam), and the pretreatment processes used (blanching or blanching and freezing), which significantly (*p* < 0.001) reduced shear forces during texture measurement. Therefore, the authors recommend shortening the SV process to 11 min, which requires the use of pre-treatment methods.

The structure of carrots is based on tissue stiffening, which is the result of a combination of factors, including cell wall composition (especially cellulose, pectin, and lignin), water content and turgor pressure, and the accumulation of structural proteins like extension. Cell wall components (especially cellulose) possess the function of maintaining the rigidity and resistance of tissues. The cell walls are glued together by the middle lamella, forming cell clusters. Cooking causes transformation and dissolution of the middle lamella substance, softening and loosening of the cellulose fibers in the cell walls, and other stiffening elements [77]. Thus, changes in texture (softening) during cooking are caused by the breakdown and release of pectins from the middle lamella, which leads to cell wall separation and the loss of stiffness.

In addition to the positive effect of increasing process parameters on the degree of pasteurization and thus the hardness of carrots [40], the literature emphasizes the importance of the dimensions of the raw material. Too large dimensions, particularly product thickness, cause uneven cooking of the product due to non-uniform heat transfer to the deeper layers [35].

The results of Koç et al. [40] indicate that the highest consumer acceptance was achieved for carrots prepared at ~75–80 °C. Increasing the process temperature resulted in an excessive reduction in hardness. Carrots prepared SV (89 °C for 30 min) were characterized by lower hardness than those boiled for 7 min [36].

Further study [28] showed that SV cooking at 80 °C required a longer process time to achieve the same hardness as cook-vide, while cooking at 90 °C produced a harder consistency than cook-vide. In the study by Rinaldi et al. [34], carrots processed by the SV method required a significantly higher (*p* < 0.001) cutting force and therefore had a higher hardness than steamed carrots. However, no statistically significant (*p* > 0.05) differences were found between SV and cook-vide carrots.

Broccoli. The hardness of broccoli florets cooked in boiling water for a maximum of 15 min did not differ (*p* > 0.05) from that of florets cooked at lower temperatures (85–90 °C), either under vacuum conditions (SV) or at normal atmospheric pressure (immersion cooking) [39]. Martínez-Hernández et al. [51] found that broccoli boiled and steamed exhibited lower hardness compared to SV broccoli (using steaming and microwave cooking). On the other hand, microwave cooking and grilling resulted in higher shear forces during texture measurement. These methods prevent the raw material from contact with the aqueous environment and the vacuum, which limits the evaporation of internal water and the transformation of pectin. Sous-vide cooking methods (in a steam chamber, in a microwave) resulted in a product with intermediate hardness compared to other methods.

Other vegetables. The hardness of vegetables (green beans, red cabbage) decreased with the change in temperature and processing time, but temperature had a greater effect. Traditional cooking, compared to the cook-vide (90 °C) and SV (90 °C) methods, allows for a product with increased softness. Preparing green peas using the SV method requires slightly longer thermal processing to achieve the same degree of softness as the cook-vide method. Similar results were obtained for carrots, potatoes, and broccoli. This may be because in the SV method, the pectin contained in the cell walls has limited access to water, which slows down the softening process [28,49,67]. It should be emphasized that the pressure used during vacuum packaging does not affect hardness [39].

The hardness of vegetables prepared using the SV method depends on the process parameters used. Lower temperatures (LTLT) often result in greater hardness, while higher temperatures and shorter times (HTST) can result in a softening of the structure [28,37,39,44,49,63]. As various authors have noted, vegetables cooked using the SV method are usually harder. Celery cooked SV maintained firmness, required twice as much chewing and longer consumption time, and had the highest compressive and shear forces, regardless of cooking time. Similarly, eggplants subjected to the SV method were found to be harder in terms of mechanical parameters [31,46], and the pumpkin had a more fibrous texture and was less soft than boiled or steamed samples [62].

Various authors do not observe differences between the SV and conventional methods, which may be due to comparable thermal conditions, similar to those used in these methods. Therefore, comparison of the SV method with other cooking methods should always take into account sample geometry, texture measurement procedures, and any pretreatments.

## 6. The Effect of the Sous-Vide Method on Vegetable Color

The effect of the SV method on color depends on the type of vegetables, or more precisely, the pigments they contain (Table 2). Many studies compare the same raw materials and only the SV method with different parameters, while others compare the SV method with other cooking methods. Therefore, the research results of many authors are difficult to compare.

The color effects are very diverse and depend on the type of pigment: chlorophylls, carotenoids, and anthocyanins react differently to temperature, pH, and oxygen conditions.

### 6.1. Vegetables Rich in Chlorophyll

Green vegetables, rich in chlorophyll, are most sensitive to color changes during heat treatment. In the case of green vegetables (broccoli, green beans, green peas, *Brussels sprouts*, asparagus), the color depends on the selected process parameters and the content of chlorophyll and pheophytin after the process. It should be emphasized that chlorophyll is easily washed out of the structures of green vegetables, especially leafy ones. Sous-vide cooking results in less conversion of chlorophylls to pheophytin (i.e., preserving the green hue) than cooking in direct contact with water, especially when this process occurs in an oxygen-limited environment. The a/b ratio is used to measure the intensity of green color. This parameter increased with increasing temperature in the initial phase of the process. This can be explained by a faster evaporation of water from the surface of broccoli. Further processing leads to chlorophyll loss and its conversion into derivatives, depending on temperature and pH [28,37,38,39,47,51,53,54,63].

**Figure 1:**
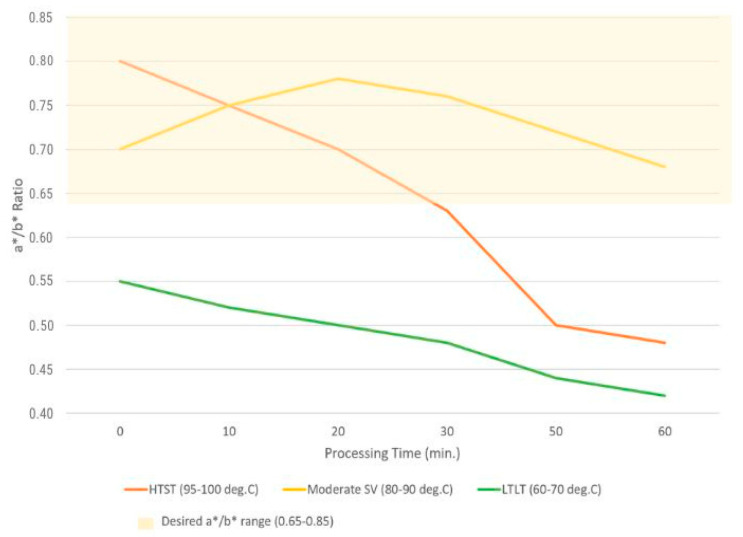
Changes in the a*/b* ratio in broccoli depending on the sous-vide processing parameters: HTST—high temperature, short time, SV—sous-vide, LTLT—low temperature, long time. Source: own interpretation based on [39,51,52,54].

The research by Pero et al. [54] indicates that a*/b* values in the range of approximately 0.65–0.85 are responsible for the most liking, intense green color of broccoli after SV treatment. These parameters are associated with short processes, i.e., 85–95 °C for 10–20 min, during which minimal chlorophyll changes occur. Processing at 60–80 °C for periods longer than 30 min gradually reduces the a/b value (below 0.60), indicating an initial loss of color intensity. However, the rate of this degradation is slower than in boiling in a pot. Therefore, to maintain an attractive color, the process time should not be extended. Peroxidase is considered the most stable enzyme during the thermal processing of vegetables. To prevent loss of the attractive color of broccoli, especially during refrigerated storage, it should be processed at temperatures above 80 °C to inactivate this enzyme. The best results are achieved by using higher temperatures (99 °C) for shorter periods [54]. High a* values (tendency toward olive green) are only observed with longer processing times and/or contact with an acidic environment. Figure 1 shows the a*/b* values depending on the temperatures and times used in the processes (HTST, SV, and LTLT) [28,37,38,39,47,51,53,54,63].

In another study [39], lowering the cooking temperature compared to the traditional method (100 °C) resulted in a simultaneous reduction in the red color changes. The use of vacuum packaging in the SV method prevents the oxidation of denatured chlorophyll. During heat treatment, which induces protein coagulation, chlorophyll is exposed to acids contained in plant tissue, making it susceptible to conversion into olive-green pheophytin. In turn, the total color change (ΔE) was greater for green peas prepared by the SV method than for those prepared by the cook-vide method. It is likely that during the SV cooking, the water in the intercellular space of the peas changes from a liquid to a gaseous state due to the high temperature and long processing time. This results in an increase in the penetration coefficient and more advanced chlorophyll degradation. The conversion of chlorophyll to pheophytin and its isomers also contributed to this change. In this case, the process temperature had a higher significance (*p* < 0.05) [40].

The color of broccoli subjected to lower heat treatment parameters (85–90 °C) did not differ from the lightness of the traditionally prepared samples. However, significant differences (*p* < 0.05) were found in the value of parameter a*. A greater decrease in the a* value was observed in broccoli cooked SV and at 85–90 °C, indicating a color change towards green. The samples were greener than the raw material itself. This is suggested to be related to a modification of the surface reflective properties caused by the exchange of intercellular air for cell sap [39]. Martínez-Hernández et al. [51] found an opposite relationship. Sous-vide and grilled broccoli had a less green color (higher values of parameter a*), while conventionally cooked broccoli resulted in a 36% decrease in a* value. A significant reduction in color tone was observed only in broccoli cooked by the SV and grilled methods. No differences in color were determined between steamed and SV samples. Similar results were obtained by Lafarga et al. [52,53].

Color changes in green vegetables are caused by chlorophyll degradation. In conventionally cooked broccoli, the total chlorophyll content was statistically significantly (*p* < 0.05) lower than in broccoli cooked at low temperatures under aerobic conditions and using the SV method. The content of chlorophyll b (green-yellow) in broccoli cooked using the SV method did not differ significantly from the content in the raw material and is lower in broccoli prepared using the other method (*p* < 0.05). Vacuum conditions, the absence of an aqueous environment, and the reduced temperature in the SV method favored smaller transformations of chlorophyll (green color) to pheophytin (olive-green color). The highest pheophytin content was found in samples cooked in boiling water (100 °C), followed by those cooked in water at 85–90 °C and SV samples [39]. According to Martínez-Hernández et al. [51], the lowest levels of chlorophyll a (62%) and b (39%) retention were found in SV broccoli, compared to other analyzed methods (cooking in water, steaming, microwave cooking, grilling).

Cooking *Brussels sprouts* using the SV method resulted in a product with a less green color than steaming [34]. This is evidenced by the statistically significantly higher value of the a* coordinate of SV *Brussels sprouts*. However, no differences were found in the lightness of the samples (L*) or the b* coordinate value.

Thermal processing of kale using the SV method resulted in less change in overall color (chlorophyll) than conventional cooking. The SV samples were also lighter. Kale cooked by SV at 85–95 °C had less blue color than traditionally cooked kale and was also less green. High SV process parameters (5–10 min, 105 °C) allowed the kale to retain a more attractive green color than lower parameters of 85–95 °C or conventional cooking [65]. According to Lafarga et al. [52,53], kale leaves cooked SV (80 °C for 15 min) were darker in color than steamed kale.

Although the total color change (ΔE) of green beans did not differ between the traditional, cook-vide, and SV methods, an increase in the a* coordinate value of the SV sample was determined compared to the cook-vide method. It was found that the main factor responsible for the color change was the processing time, despite the temperatures used exceeding the threshold for inactivation of oxidative enzymes in green vegetables [27,28].

### 6.2. Vegetables Rich in Carotenoids

Carotenoids are relatively resistant to heat treatment, but their visibility may vary depending on secretion from damaged cell structures. The values of the L and a parameters remain close to those of the raw material even after SV treatment, and the most favorable results are obtained at a temperature of 80–90 °C and a time of 20–30 min. Anthocyanins, on the other hand, are characterized by the highest stability in vacuum conditions; therefore, SV treatment favors their retention and more intense purple-red colors. The contradictory results obtained by different authors may be due to differences in process parameters (e.g., process duration), raw material pH (which impacts dye stability), water content, and the presence of metal ions, as well as the color measurement technique (instrumental Lab* vs. sensory evaluation). Changes in the a* parameter should be interpreted in terms of the consumer’s perception threshold—a small change in the instrumental change parameter does not always mean a noticeable difference to the consumer, especially when the texture or aroma improves at the same time [28,37,38,39,47,51,53,63].

The color of carrots prepared by the SV method did not differ significantly from the color of steamed carrots [34]. Koç et al. [40] found no differences in the total color change (ΔE) between SV and cook-vide cooked samples, despite the significantly longer SV processing time. There were also no statistically significant differences in the b* coordinate (blue-yellow) between the SV and pot-boiled in water samples. In the study by Guillén et al. [39], it was found that carrots cooked by the SV method had a higher a* coordinate value, while lightness did not differ between samples.

A study by Iborra-Bernad et al. [28] showed that carrots cooked at 100 °C for 10 min exhibited a statistically significant (*p* < 0.05) lower change in color (ΔE) than carrots cooked SV (90 °C, 30–60 min) and cook-vide (80 °C, 40–70 min). The SV processing time does not seem to affect the color of carrots at high parameters such as HTST (high temperature, short time) [37], while in LTLT (low temperature, long time) (60 °C for 72 h), it significantly (*p* < 0.05) reduces the L*a*b* parameter values compared to the raw material [35].

Carotenoids, the pigments contained in carrots, are quite resistant to the cooking process. Although higher amounts were detected in carrots prepared by the SV method [38,39], the β-carotene content was similar to that in the raw material and lower than in samples cooked using conventional methods. This may indicate easier extraction of this compound from damaged cellular structures.

### 6.3. Vegetables Rich in Anthocyanins

The color of other anthocyanin-rich vegetables (red cabbage, purple potatoes) seems to be better preserved after SV processing compared to other methods, as evidenced by a higher value of the a* coordinate and a statistically significantly higher (*p* < 0.05) anthocyanin content [67]. Red cabbage prepared using the SV method contained twice as many (*p* < 0.05) anthocyanins as samples cooked in water [67]. No differences were observed in the color of vegetables rich in anthoxanthins, such as cauliflower [33,52,53].

The SV and steam cooking caused a lower and higher difference in color parameters in cooked chicory stems compared to the other cooking methods. Likely, the low oxygen presence and efficient heat transfer to the vacuum-packed vegetable in SV cooking reduce the color modifications caused by enzymatic and non-enzymatic reactions [68].

## 7. The Effect of the Sous-Vide Method on Vegetable Taste and Aroma

Available sensory data indicate a frequent increase in the acceptability of some vegetables prepared SV (e.g., samples of green peas or red cabbage). These ratings depend on the type of vegetable and the process parameters. Sous-vide often protects volatile aromatic compounds from oxidation due to the vacuum conditions, which translates into a more intense “freshness” profile (e.g., terpenes in carrots). On the other hand, enhancing the flavor of some vegetables may lead to overly intense flavor notes for some consumers.

### 7.1. Potatoes

Kowalewicz et al. [33] showed that potatoes prepared using the SV method were more acceptable compared to those cooked by steaming and boiling. The attractiveness of overall appearance, taste, and texture was highlighted, though these differences were not statistically significant (*p* > 0.05). The addition of rosemary oil to vacuum-packed potatoes intended for cold storage and subsequent SV processing has been shown to minimize the detection of foreign flavors and odors [78], while also protecting against color change during storage [79].

### 7.2. Carrots

Vacuum conditions protect volatile compounds from oxidation, as shown by a study on carrots [34]. Sous-vide samples did not differ statistically significantly *(p* > 0.05) in terms of terpene composition from raw carrots, and contain more α-pinene (woody and pine notes) and terpinolene (fresh, sweet, pine, citrus notes) levels. A decrease in terpene content was determined only in the amount of β-myrcene (peppery notes, sharp notes). Greater changes compared to the raw material were found in the main groups of volatile compounds in carrots: aldehyde levels were similar to those in steamed carrots, and alcohol levels were lower in SV carrots. The content of esters and ethers also decreased. Although the fraction of terpenes responsible for freshness and “green” notes decreased significantly (*p* < 0.05) during storage, their levels at the end of the storage period were still higher than in steamed carrots [34].

A study conducted by Araya et al. [41] found that carrots prepared using the SV method, like raw carrots, contained more ethanol and had more earthy notes during storage. They also exhibited a more intense flavor and a less intense odor than carrots cooked in water. In both cases, carrots cooked using the SV method had a flavor and aroma profile similar to the raw material. An increased perception of the “green” flavor and sweet taste was observed. Carrots cooked in water had a more intense flavor and aroma of overcooked vegetables, which consumers are accustomed to. Sous-vide cooking was also associated with greater carrot hardness and force required to cut the carrots compared to boiling, resulting in a crunchier texture and longer chewing time [41]. Due to the lack of direct contact with the aquatic environment, SV carrots were also less juicy, which was reflected in instrumental measurements.

Moreover, Kowalewicz et al. [33] observed no statistically significant (*p* > 0.05) differences between the acceptability of smell, texture, taste, or overall acceptability of SV carrots, steamed carrots, and boiled carrots. In the study by Iborra-Bernad et al. [36], a greater number of consumers (*p* < 0.05) preferred SV carrots in terms of taste acceptability compared to cook–vide carrots; no such differences were found for boiled carrots.

### 7.3. Other Vegetables

The volatile compound profile also varied in *Brussels sprouts*. The greatest changes were observed in the content of nitrile compounds (e.g., butanenitrile), which impart a garlic aroma, and were present in higher amounts in SV *Brussels sprouts*. Sous-vide tests also showed significantly (*p* < 0.05) higher losses of sulfur compounds (thiocyanates and isothiocyanates). Their lower content leads to the formation of appetite-stimulating compounds and a pleasant, mild aroma [34].

Chicory prepared using the sous-vide method was characterized by significantly (*p* < 0.05) higher acceptance of taste, aroma, and overall quality. No significant differences were found in the acceptance of chicory texture; however, the highest values were found in the SV method, which did not differ significantly (*p* > 0.05) from steaming. Similarly, the color, although the highest in the SV method, did not differ significantly (*p* > 0.05) from the acceptability of chicory boiled. No differences were found in the content of soluble sugars (glucose, fructose, sucrose, and total sugars) [80].

The acceptability score of asparagus samples prepared using the SV method did not differ statistically significantly (*p* > 0.05) from those cooked in water and in a microwave oven; however, the authors emphasize that the acceptability of the SV method samples (and especially those prepared in a microwave oven) was statistically significantly higher (*p* < 0.05) [1].

A significantly higher (*p* < 0.05) sensory quality of SV samples was demonstrated in terms of texture, taste, and overall palatability compared to cook-vide and traditionally cooked samples [49]. Moreover, consumers (n = 92) in the Iborra-Bernad study [67] indicated that red cabbage cooked by the SV method was more preferred in terms of hardness, odor, and taste compared to samples boiled in water.

Higher nutrient retention in vegetables prepared SV is associated with the intensification of vegetable-specific flavors, and it may cause some vegetables (e.g., turnip and rutabaga) to have a flavor that is too intense for individuals [3]. Better taste and overall acceptability were also found in green peas prepared using the SV method [75], while the overall acceptability of potatoes, carrots, and parsley did not differ significantly (*p* > 0.05) [33].

## 8. The Effect of Sous-Vide Heat Treatment on the Nutritional Value, Bioavailability, and Digestion of Vegetables

The effect of the SV method on the content of folates, vitamin C, and antioxidant activity depends on the raw material. The content of phenolic compounds and vitamin C in vegetables appears to be higher in SV samples compared to conventional methods, and often comparable to or higher than those in steamed samples. In turn, most studies confirm the higher antioxidant activity of vegetables prepared using the SV method. Although the mineral content depends on the raw material, it is similar to or even higher than that in steamed vegetables. During cooking, these minerals often transfer to the broth. The content of glucosinolates in cruciferous vegetables is generally lower in samples cooked by the SV method compared to boiling in a pot or steaming [58].

Due to the large variation in the results obtained depending on the type of raw material, the effect of the SV processing method on selected vegetables is presented below.

### 8.1. Potatoes

The water content in potatoes is reflected in the content of bioactive substances. According to Ibbora-Bernad et al. [27], the less intensive parameters of the SV method allow for higher anthocyanin retention in potatoes compared to other methods. Other authors [61] indicated no differences in the antioxidant potential, expressed by DPPH and FRAP values, between potatoes boiled and cooked SV [61].

### 8.2. Carrots

Study [38] showed that SV carrots contained higher levels of carotenoids, phenolic compounds, and ascorbic acid than steamed carrots. Moreover, there was only a slight decrease in phenolic compounds during storage. This is confirmed by the results of Guillén et al. [39], which showed that carrots prepared by the SV method contained statistically significantly (*p* < 0.05) more carotenoids and had higher phenolic compound retention and antioxidant activity than samples cooked at temperatures below 100 °C and in boiling (conventionally).

In a study conducted by Iborra-Bernad et al. [28], the content of β-carotene in SV samples (80–90 °C) did not differ (*p* < 0.05) from that in raw carrots, while carrots cooked in water and the cook-vide method, which had direct contact with the aqueous environment, contained statistically significantly (*p* < 0.05) more β-carotene. This is probably due to the higher process temperature and its effect on destabilizing cellular homeostasis. It facilitates the disintegration of carotenoid-protein complexes and leads to higher extraction of β-carotene. This may indicate less damaged cellular structure in products prepared by the SV method.

In turn, the antioxidant potential (significantly higher DPPH and FRAP values, *p* < 0.05) was higher in carrots cooked using the SV method compared to those boiled in a pot [61]. Similarly, the study by Patras et al. [43] demonstrated that carrots cooked using the SV method had higher antioxidant activity than those cooked in water. On the other hand, the phenolic compounds in SV samples decreased by 29.2%, while in water-cooked samples, they remained at the same level as in the raw material. This was because the carrots cooked SV were subjected to a higher heat dose (90 °C, 10 min) than those cooked in water (70 °C, 2 min), and were blanched before packaging. The total phenolic content of carrots cooked in water decreased significantly (*p* < 0.05) after 5 days of storage (by 83%), while in SV carrots it remained at 57% even up to the 20th day of refrigerated storage. Similar variations were observed for carotenoids in the stored carrot samples. Carotenoid loss in carrots cooked by SV was only 4.1%, while those boiled lost 42.9% [43].

The effect of various heat treatment methods, i.e., boiling, steaming, steaming in a conventional steam oven, baking in a combi steamer, and SV, on the total phenolic and flavonoid content, phenolic and carotenoid profiles, and antioxidant capacity was assessed. Among the heat-treated samples, the lowest TPC value (1.01 mg GAE/g dry weight) was found in carrots cooked using the SV method. This sample did not differ significantly (*p* > 0.05) from raw carrots. Total phenolic acid content in raw carrots was 1718.18 µg/g dry weight, compared to 1744.68 µg/g dry weight in SV-cooked carrots, with no statistically significant difference (*p* > 0.05) between these samples. The SV technique caused the smallest changes in the content of phenolic compounds and carotenoids, as well as antioxidant potential, compared to raw carrot roots. The authors suggest further research on the effect of SV cooking temperature on bioactive compound profiles and sensory properties [42].

The authors [40] emphasize that the degree of degradation of antioxidant activity in carrots prepared by the SV method increased with the increase in processing time and temperature. Losses were lower in the SV method (13.3–34.9%) than in cook-vide (18.6–51.7%). The degradation of phenolic compounds in carrots prepared by the SV method ranged from 8.9 to 20.4%, depending on the process temperature (*p* < 0.05), and did not differ significantly (*p* > 0.05) from cook-vide carrots (4.4–19.3%). Moreover, carrots prepared using the SV method had 10.8–36.2% less vitamin C than the raw material, and cook-vide carrots had 5.7–27.4% less. These results did not differ in terms of statistical significance (*p* < 0.05); in both cases, the retention level was influenced by the process temperature.

### 8.3. Broccoli

Guillén et al. [39] found that the SV method allows for very high retention (89.9%) of phenolic compounds in broccoli, statistically significantly (*p* < 0.05) higher than in broccoli cooked in water at 85–90 °C (~76%) and 100 °C (42%). A similar trend was observed during the analysis of antioxidant activity, although the retention levels were much lower (SV—~50%; cooking < 100 °C—~34%; conventional cooking—~30%).

A study conducted by Czarnowska-Kujawska et al. [50] compared various cooking methods: SV, boiling in water, steaming, and microwave cooking. Broccoli and spinach cooked SV retained folate levels up to 70–80% compared to the raw material, with less loss than when boiled in a pot. The SV method preserves folates better than boiling; folate losses did not exceed 30% [50].

In a study where SV cooking was not preceded by blanching, the content of phenolic compounds in SV broccoli was found to be higher than that in broccoli cooked in water and at a similar level to that of steamed broccoli [51]. The lower content of phenolic compounds in SV broccoli, compared to grilled and microwaved broccoli, may be due to the longer processing time and the use of a vacuum, which causes cell destruction and promotes the leaching of phenolic compounds. However, during grilling, compounds are formed that can react with Foline Ciocalteu reagents, inflating the analytical results. The SV method, microwave cooking, and their combination resulted in higher antioxidant activity of broccoli. Broccoli cooked using the SV method has statistically significantly (*p* < 0.05) higher DPPH (1,1-diphenyl-2-picrylhydrazyl) and FRAP (Ferric Reducing Antioxidant Power) values compared to steaming, grilling, and pot cooking [51].

A study by Lafarga et al. 52,53] found no differences in the content of phenolic compounds, vitamin C, and FRAP between steamed broccoli (100 °C, 15 min) and SV-processed broccoli (80 °C, 15 min). The authors [52,53] also found that the content of antioxidant components varied depending on the morphological part (stem, floret) and broccoli cultivar. Although the processing method used (steaming and SV) did not affect vitamin C content in the samples, significant differences (*p* < 0.05) were identified in the content of phenolic compounds, DPPH, and FRAP. This supports the conclusion that steamed broccoli contains higher levels of these compounds in the floret, while SV-processed broccoli retains more in the stem.

The higher antioxidant potential of SV broccoli is supported by the results of Kosewski et al. [61], who reported that these samples had statistically significantly higher (*p* < 0.05) DPPH and FRAP values compared to conventionally cooked broccoli (in water). However, Florkiewicz et al. [59] found that SV broccoli had higher vitamin C content (*p* < 0.05) than broccoli boiled or steamed. In turn, phenolic compounds levels and antioxidant activity (ABTS index) were comparable to steamed samples and higher than in broccoli boiled. Glucosinolates (GLS) are sulfur- and nitrogen-containing secondary plant metabolites commonly found in cruciferous vegetables. Scientists are interested in GLS due to its anti-cancer properties. Their presence in cruciferous vegetables determines their characteristic flavor and aroma. According to Florkiewicz et al. [58], broccoli processed by the SV method contained significantly less (*p* < 0.05) glucosinolates than steamed broccoli, but significantly more (*p* < 0.05) than boiled broccoli. Similar relationships were observed after 5 days of refrigerated storage. It should be noted that higher GLN content after SV processing, compared to cooking in water, was observed only in broccoli.

In the study, Florkiewicz and Berski [60] highlight the positive effect of the SV method on mineral retention, which is higher than that of steaming. Analysis of the presented data suggests that the content of micronutrients (Mn, Fe, Zn, Cu) and macronutrients (K, Na, Ca, Mg) in cruciferous vegetables is comparable to the content in the steaming process. Cooking in a pot with water causes greater losses. Analysis of the limited data on changes in the mineral content of broccoli after heat treatment supports the effectiveness of steaming, although micronutrient levels remain similar in both methods. The highest copper content was found in broccoli and other cruciferous vegetables tested using the SV method.

### 8.4. Cauliflower

The study by Florkiewicz and Berski [60] showed that SV cauliflower contained statistically significantly (*p* < 0.05) more potassium and manganese than steamed samples. While sodium, calcium, and zinc levels were higher than in steamed samples, these differences were not statistically significant (*p* < 0.05). Different trends were observed in Romanesco cauliflower, which suggests that mineral retention depends on the raw material and its variety. These and other studies [59] indicate that the content of bioactive compounds depends on the variety. Cauliflower prepared by the SV method contained more phenolic compounds than steamed cauliflower, while antioxidant activity and vitamin C content were similar (*p* > 0.05). In the case of Romanesco cauliflower, SV cooking resulted in significantly higher (*p* < 0.05) vitamin C and phenolic compound contents than steaming and boiling. Antioxidant activity did not vary depending on the preparation method.

Kosewski et al. [61] highlighted the higher antioxidant potential (*p* < 0.05) of SV cauliflower compared to immersion-cooked cauliflower. According to Lafarga et al. [52,53], there are no differences in the content of phenolic compounds, vitamin C, and FRAP between SV and steamed cauliflower samples.

It was also found that the levels of caffeic, p-coumaric, and gallic acid were most stable in vegetables prepared by SV. The glucosinolate content in common and Romanesco cauliflower cooked using the SV method was statistically significantly lower (*p* < 0.05) than in cauliflower cooked conventionally in water or by steaming [58].

### 8.5. Brussels sprouts

*Brussels sprouts* processed by the SV method contained less glucosinolates (*p* < 0.05) than those cooked by steaming and boiling [58]. It was also found that *Brussels sprouts* prepared using the SV method were characterized by a statistically significantly higher (*p* < 0.05) vitamin C content, while phenolic compound levels remained similar to those cooked in water (*p* > 0.05), and antioxidant activity (ABTS) was similar to that in those steamed (*p* > 0.05) [59]. Mineral content in *Brussels sprouts* prepared by the SV method, such as potassium (*p* > 0.05), sodium (*p* < 0.05), magnesium (*p* > 0.05), iron (*p* < 0.05), and copper (*p* < 0.05), was statistically significantly higher or at a similar level to that in steamed samples. The content of these compounds in samples boiled was usually lower, although calcium and manganese levels did not differ among the samples [60]. Some studies indicate that glucosinolates in *Brussels sprouts* may be lost during SV cooking. However, the overall retention of other beneficial compounds remains high, and it is more effective than other cooking methods in maintaining antioxidant, polyphenol, and vitamin C levels in various vegetables [55,59,60].

Conventional cooking resulted in the greatest loss of dry matter content due to leaching of components into the aqueous medium, while SV and steaming methods retained dry matter levels comparable to raw *Brussels sprouts*. Sous-vide cooking retained the highest protein content, while boiling resulted in the greatest protein loss. This loss during boiling is due to both denaturation caused by high temperature and partial leaching of water-soluble protein compounds. Additionally, the fat concentration was maintained at a similar level to raw *Brussels sprouts*, ensuring the highest fat retention. The fiber content decreased, but to a lesser extent than during cooking in water. The SV method was considered the best method for maintaining MUFA content, as well as the mineral values of potassium, sodium, magnesium, iron, and zinc, at consistent levels comparable to raw *Brussels sprouts*. After in vitro digestion, the content of individual minerals decreased significantly *(p* < 0.05). The highest bioavailability of magnesium was found in *Brussels sprouts* cooked SV and in water, while for calcium, boiling resulted in the highest bioavailability. However, raw *Brussels sprouts* demonstrated the highest bioavailability of potassium and zinc [55,56].

The effect of various hydrothermal processes and subsequent in vitro digestion on the availability of selected bioactive compounds, in particular glucosinolates (GLS), isothiocyanates, and indoles, in *Brussels sprouts* was investigated. The SV method leads to significantly (*p* < 0.05) higher losses of glucosinolate compared to conventional cooking methods. The glucosinolate digestion process was found to be more efficient following the SV method. Specific glucosinolates, namely glucoraphanin (GRA), glucoraphenin (GIV), and gluconasturtin (GNS), were completely degraded during digestion. The study highlights that although SV cooking may lead to greater glucosinolate losses, the subsequent digestion process may be more efficient for these compounds after SV and conventional cooking. Some glucosinolates are completely degraded, while others, such as methoxyglucobrassicin, show resistance [81]. Additionally, the SV method proved to be the most effective, demonstrating excellent retention of polyphenolic compounds and high antioxidant activity. This suggests that SV is a better alternative to steaming for preserving these beneficial properties. The effect of this method on the concentration of bioavailable polyphenolic compounds was largely maintained even after in vitro digestion, except for antioxidant activity [55,56].

### 8.6. Kale

Armesto et al. [65] investigated the effect of SV cooking parameters on the quality of kale. Cooking kale SV (85–95 °C, for 30–90 min) resulted in higher losses of soluble substances and vitamin C, and lower losses of phenolic compounds than cooking in water (105 °C, 5–10 min). The use of high process parameters was associated with higher antioxidant activity and retention of phenolic compounds and chlorophyll than SV cooking at a lower temperature and for a longer duration [65]. Kosewski et al. [82] also indicated higher antioxidant activity in kale cooked using the SV method (84 °C, 30 min) compared to conventionally cooked samples.

When comparing the SV and steaming methods, the SV method is preferred due to lower losses of vitamin C and antioxidant activity than steaming [52,53]. These authors did not find such differences in the content of phenolic compounds in kale. Green peas.

The degradation of antioxidant activity in green peas depended on the set process parameters. Both increasing temperature and heating time in the SV method had a significant (*p* < 0.05) effect on the level of losses. In the cook-vide (CV) method, temperature played a more important role. Lower levels of antioxidant activity losses were determined in the sous-vide method (15.9–46.0%) compared to the CV method (1.7–71.2%). Similar relationships were found for vitamin C losses of 20.7–43.8% (SV) and 11.4–58.1% (CV), whereas phenolic compound losses were 13.7–20.7% (SV) and 2.3–12.3% (CV), respectively [49].

### 8.7. Other Vegetables

No effect of heat treatment methods (SV, boiling in water, steaming, and microwave cooking) on the content of calcium, magnesium, and nitrates in chicory was observed. The sodium and potassium content was statistically significantly lower (*p* < 0.01) only in samples boiled in water. The total phenol content was significantly higher (*p* < 0.01) in chicory cooked in a microwave oven, while SV cooking generally did not differ (*p* > 0.01) from boiling and steaming. In the case of one variety, statistically significant (*p* < 0.01) differences were found to the detriment of chicory cooked in water, which was reflected in the antioxidant activity [68].

The polyphenol profile and antioxidant potential of five raw vegetables (beetroot, red cabbage, red pepper, green pepper, and kale) were determined after steaming and SV cooking at different temperatures (80 °C, 85 °C, and 90 °C). Total polyphenol content was determined spectrophotometrically using the Folin–Ciocalteu reagent, antioxidant properties were assessed using the DPPH radical, and the polyphenol profile was evaluated by the HPLC–UV–VIS method. The SV method at 85 °C produced the best results, with the lowest losses or the highest increases in total polyphenol content, while the SV methods at 80 °C and 85 °C had the same effect on antioxidant potential and polyphenol profile [66].

The SV cooking method has a positive effect on the digestibility of vegetables, their nutritional value, particularly their mineral content, and bioactive ingredients. Sous vide cooking preserves essential minerals such as potassium, magnesium, calcium, and phosphorus levels comparable to raw vegetables [16].

The effect of different cooking techniques (boiling, pressure cooking, SV, and cook-vide) on the physicochemical and bioactive properties of ready-to-eat vegetable and lentil soup was investigated. The bioavailability of these compounds was assessed using an in vitro gastrointestinal simulation method. The bioavailability of phenols was higher in the raw sample and then the conventionally cooked, SV, cook-vide, and under pressure (*p* < 0.05) [83].

Research highlights that steaming and SV cooking methods result in better retention of nutrients and beneficial compounds compared to conventional cooking (Table 3).

## 9. The Effect of Sous-Vide Heat Treatment on the Microbiological Quality of Vegetables

Few studies have examined the microbiological quality of vegetables prepared using the SV method and stored under refrigerated conditions. Although SV is sometimes used as a pasteurization step, the reduction in microbial load may not be sufficient to prevent product spoilage throughout its shelf life [84].

Sous-vide cooking has shown significant (*p* < 0.05) inactivation of pathogens such as *Salmonella* (4.34 log), *Campylobacter* (4.82 log), and *Clostridium* spores (1.70 log) when cooked at the appropriate temperatures [85]. Studies indicate that although vegetative cells are generally inactivated, spores may survive, requiring further evaluation of the safety of long-term storage [85,86].

However, SV heat treatment combined with appropriate packaging methods has been found to effectively maintain the microbiological safety of vegetables such as red peppers (*Capsicum annuum* L.), keeping the total viable count below 4 log CFU/g for up to 24 days during refrigerated storage, thereby extending shelf life and ensuring safety. SV heat treatment significantly (*p* < 0.05) reduced the initial microbial load of red bell peppers compared to control samples. In particular, it reduced total viable counts (TVC) to less than 2 to 2.35 log CFU/g, *Enterobacteriaceae* to less than 2 log CFU/g, and microscopic filamentous fungi to less than 2 log CFU/g. In control samples, TVCs ranged from 3.54 to 3.86 log CFU/g. While raw peppers typically have a shelf life of 3 to 6 days, sous vide-processed peppers (60 °C for 30 min and 70 °C for 15 min) can be stored for 15 to 24 days, depending on the heat treatment and packaging method used [87]. The microbiological safety of vegetables prepared using the SV method is largely based on precise time and temperature control during cooking and subsequent cooling. Extended cooking times at specific temperatures can increase pathogen inactivation, but improper handling may pose safety hazards [23].

In the study by Martínez-Hernández et al. [51], found that broccoli prepared by the SV method and stored for 45 days was characterized by an average growth rate of mesophilic and psychrophilic microorganisms, similar to that observed during broccoli steaming. The highest growth was observed during microwave cooking (including SV in a microwave oven) and boiling in water. The lowest growth rate was recorded during grilling (process temperature 280–300 °C). The microbiological quality of *Brussels sprouts* prepared using the SV method was higher than in samples cooked by steaming. In the case of carrots, both methods led to the inactivation of microorganisms to a level of <1 log CFU/g [34].

In another study, the microbiological and physicochemical stability of broccoli prepared by the SV method was monitored for 90 days under standard (4 °C) and slightly aggressive (6–10 °C) storage conditions. Microbiological safety was maintained under all conditions, with constant water activity and only moderate acidification [88].

Another study found that both the control and high-pressure SV green beans showed no bacterial growth during 60 days of refrigerated storage, indicating strong microbiological safety due to the effective cooking treatment at 90 °C for 1 h and 45 min [89].

Sous-vide vegetable and beef mixtures are safe to consume for up to 45 days when stored in the refrigerator. After cooking, the dishes were immediately cooled to 5 ± 0.5 °C and stored at 2 ± 1 °C for up to 45 days. Microbiological and physicochemical analyses were performed immediately after processing and after 7, 14, 21, 30, and 45 days of storage. The dishes met the microbial standards for mixed products. The physicochemical analyses only showed differences in color parameters (L*, a*, and b*) depending on the storage time in each analyzed dish [90].

While the SV method has its advantages in preserving nutrients and extending shelf life, it may not be sufficient on its own to guarantee complete safety from all microbiological hazards, such as spores. Further research is needed to investigate additional safety measures, such as combining SV cooking with non-thermal methods to improve food safety [14].

The combination of SV and microwave cooking (SV–Mv) results in the effective reduction in mesophilic bacteria, yeasts, and molds for up to 30 days under cold storage. This method allows for a rapid reduction in the number of *E. coli* and *L. monocytogenes* in chicory by more than 5 log CFU/g [80]. The extension of microbiological stability of green pea paste up to 14 weeks of storage in a refrigerator was demonstrated by Kirse et al. [91].

The authors also suggest using natural inhibitors and essential oils as methods to increase microbial safety when using the SV technique [14]. Amoroso et al. [30] confirmed that the SV packaging method combined with rosemary essential oil (REO) is an effective strategy for the qualitative preservation of potato slices during refrigerated storage for up to 11 days. In another study [92], the use of essential oil from *Elettaria cardamomum Maton* var. minuscula in SV-prepared vegetables demonstrated strong antimicrobial activity against several bacteria and yeasts, with minimum inhibitory concentrations ranging from 0.33 to 0.56 mg/mL.

## 10. Consumer Acceptance of the Sous-Vide Method

Consumers are seeking both healthy eating options, particularly transparency about food origins, and new gastronomic experiences, such as SV dishes [23].

Avató et al. [93] focused on consumer preferences for ready-to-eat (RTE) vegetable meals prepared using the SV method. Consumer perceptions of the quality, convenience, and nutritional value of such products were analyzed. The results demonstrated consumer interest in premium RTE meals, particularly those produced using the SV method. Consumers expect products that are convenient to use, high-quality, natural-tasting, and minimally processed. The SV method is perceived as a method that combines high culinary quality with consumer convenience. Consumers accept products prepared using the SV method. The acceptance of ready-made meals was influenced by high quality (95% of responses), ease of preparation (78%), time savings, ease of use for people without culinary skills (87%), and good market availability (89%). The strongest predictors of acceptance were taste (*p* < 0.001 for the burger), texture, and appearance on the plate, rather than appearance in the package, which may suggest the need for improved SV packaging design. Moreover, consumers believe that high product quality (95%) and market availability (89%) are key factors when making a purchasing decision of SV products. Consumers would buy SV products, but they need product information, additional education, and greater market availability [94]. According to Roascio-Albistur and Gámbaro [95], most consumers are not familiar with the SV technology, do not know what it is, or what benefits it brings. However, they are wary of cooking “in a bag” and associate these dishes primarily with highly processed foods. Similar results are reported by Grzesińska et al. [96]. Other studies on SV burgers shown improved perceptions of practicality and reduced concerns about sensory quality, which increases the product’s purchasing potential. However, consumers have expressed concerns about functional additives because they associate the SV method with processing rather than naturalness and health [97].

Research suggests that SV products appeal to consumers who want to eat healthily but do not like to cook or t have no culinary skills. Consumers accept the higher price of ready-made SV meals, appreciating the time savings (78%). At home, sous-vide products are very highly accepted, particularly for their convenience, quality, health benefits, and time savings—and consumers demonstrate a strong willingness to purchase, making SV a promising technology for the RTE market [93]. Doniec et al. [57] emphasize that modern consumers are increasingly aware of the nutritional value of food, which influences their preferences for products such as those processed SV.

## 11. Summary

Table 4 summarizes the considerations regarding the influence of SV processing on vegetable quality. Research indicates that the quality of many vegetables processed using SV is better than that prepared by other cooking methods.

This method preserves the crispness, intense color, and natural integrity of vegetables such as carrots, broccoli, and green beans, which is often impossible to achieve through traditional boiling or steaming. The SV method ensures that these vegetables are thoroughly cooked while retaining their nutritional value, making them both visually appealing and rich in essential vitamins and minerals. Results of the study show that SV improves the texture, color, and nutritional profile of vegetables, increasing their overall palatability. Broccoli, regardless of the temperature and duration of the process, retains its color, texture, and nutrients compared to conventionally cooked vegetables [45].

Sous-vide cooking produces vegetables of high sensory quality, with improved texture, higher fiber content, and freedom from pathogens. Thanks to the repeatability of the process and the extended shelf life, which ensures microbiological safety in the food service industry, it prevents food waste in the event of overproduction. Dishes can be used the next day or several days after preparation. Now, reducing food waste is a key element in building sustainable food service models. As Avató and Mannheim [93] emphasize, in the life cycle assessment of food products, the stages of preparation and final waste management generate a major part of the environmental burden. The use of SV technology can be an effective tool in reducing food losses, especially in the case of vegetables, which are most susceptible to quality loss and damage during processing using conventional methods.

Additionally, research on consumer behavior during the COVID-19 pandemic, where hygiene during food preparation was of particular importance, suggests that SV technology can also meet the growing demand for microbiological safety and portion control. The SV method addresses many needs arising during this difficult period: it helps reduce food waste through precise portioning and the long shelf life of vacuum-packed products, and it improves and maintains hygiene standards. The popularization of this technology could therefore support sustainable and food-safe practices in the future [94].

Moreover, preparing meals in a single portion under vacuum conditions can be an attractive solution for the growing number of single-person households, reducing both overproduction and food waste. The development of SV technology allows for the production of products with high sensory quality and extended shelf life [95].

As indicated by Głuchowski et al. [18], the total energy consumption during SV heat treatment of chicken was approximately 5–10 times higher than that of conventional methods (steaming, boiling). Most of the energy costs were related to heating the water bath. For example, the energy consumption for cooking one portion of chicken breast was 0.057 kWh, and for steaming it was 0.116 kWh. The total energy consumption for the SV method increased with increasing temperature from 64 to 75 °C (0.548–0.743 kWh). However, the energy consumption related solely to cooking the chicken (without taking into account heating the water bath) was 0.017–0.025 kWh. Interestingly, conventional cooking (approximately 20 min) turned out to be more energy-intensive than vacuum packaging and the hour-long heating at 64 °C in the SV method.

Research indicates that SV processes take longer than conventional cooking and can consume 3 to 10 times more energy, depending on the device, batch size, and process parameters [98]. Long processing times and the need to maintain a stable temperature significantly increase energy consumption. However, research emphasizes that the high energy consumption of SV processes applies primarily to home applications. On a catering scale, energy consumption per one portion of product is significantly lower thanks to simultaneous cooking of multiple portions, which optimizes equipment operation (continuous operation instead of frequent heating and cooling), reduces product losses (up to 20–40% less waste compared to conventional boiling, depending on the raw material), and reduces the need for re-preparation due to consistent quality. Furthermore, it is recommended to use high temperatures and short cooking times rather than low temperatures and long cooking times. The use of parameters such as heating at 85–95 °C for 5–30 min or 90–100 °C for a few to several minutes results in rapid stabilization of the product. At the same time, the color (e.g., broccoli, kale), aroma, high process yield, and microbiological safety are preserved. Moreover, applying a temperature of 60–75 °C for 6–48 h allows for gradual modification of pectins and cell structure, uniform softening of texture, and high retention of nutrients and aroma, especially in the case of fibrous vegetables and hard root vegetables [3,20,84]. It has been shown that conscious choices of processing (method, equipment, technique) can reduce energy consumption, which is important for consumers and mass production. Energy requirements vary significantly depending on the method, device, portion size, and method of use. According to the authors, conventional cooking (approximately 20 min) may be more energy-consuming than vacuum packaging and an hour of heating at 64 °C using the SV method, depending on the number of portions being prepared, which helps compensate for the energy consumption associated with heating the device [18,98]. At the same time, SV significantly extends shelf life, which reduces food waste and ensures the process’s repeatability. SV reduces losses of raw material and finished products through greater mass and nutrient retention (reducing water and volatile component losses), significantly longer microbiological stability, which also improves logistics and storage efficiency, and precise processing that minimizes burning, drying, or overheating of food. SV also allows the use of vegetables with a shorter shelf life, thanks to prior processing.

To synthetically organize the information contained in the literature, a conceptual SWOT analysis model was used, which provides a qualitative synthesis of the most frequently reported “strengths” and “weaknesses”, “opportunities”, and “threats” associated with the use of SV technology in vegetable processing (Table 5). The table elements were distinguished based on recurring themes identified in the analyzed publications, and their assignment to individual categories is interpretive in nature. The table is a qualitative conceptual synthesis developed based on the analyzed publications. Individual SWOT elements were assigned to categories based on the dominant interpretation presented in the literature.

A particular advantage of the SV method for vegetables is the preservation of color, aroma, and nutritional value—key features of modern consumers. The greatest threats remain the low awareness of the technology among chefs and consumers, the consumer syndrome “boil in the bag,” and the high implementation costs.

Future research should therefore focus on quantitatively assessing the impact of implementing SV technology with plant material in food service on reducing food waste. An interesting approach could be to consider unpredictable demand, especially in restaurants where production decisions are made based on cash register reports. Planning SV meals could be the solution. Moreover, an analysis of individual consumer attitudes towards ready-made meals prepared using the SV method could indicate the potential of this technology in the context of new eating habits and demographic changes in smaller households.

## 12. Conclusions

Heat treatment partially degrades internal phytochemicals and denatures endogenous antioxidants. However, appropriate cooking methods preserve and protect these phytochemicals and their biological properties. Preparing vegetables using the sous-vide method is a great step forward in the development of modern gastronomy, combining technological precision with high nutritional quality. Compared to traditional cooking methods, such as boiling or steaming, it enables the preparation of vegetable dishes with higher sensory and technological quality.

Vegetables prepared using the SV method retain a more intense color, natural aroma, firm texture, and higher levels of nutrients, including vitamin C, folates, phenolic compounds, and carotenoids. This process reduces mineral leaching and water loss, making the products juicier, more flavorful, and visually appealing. The SV method also provides better preservation of the cellular structure of vegetables, translating into their tenderness and longer shelf life. Due to the mild thermal conditions, this method is particularly beneficial for preparing dietary and easily digestible dishes, making it suitable for the elderly or those with special nutritional needs.

Microbiological safety is also a crucial element. Vacuum packaging limits the growth of aerobic microorganisms, while precise control of temperature and processing time enables the effective inactivation of pathogens and enzymes that cause food spoilage. As a result, products prepared using this method have a longer shelf life, microbiological stability, and a high level of food safety, which is particularly important in professional and mass catering.

Based on the analyses included in this review, it is possible to identify situations where this method offers clear advantages: when the aim is to maximizes the retention of dyes and volatile aromatic compounds; when a uniform texture and reduced water loss, as well as resource savings are important; or when preparing meals for people with special nutritional needs (geriatric patients, those with swallowing disorders), where in combination with appropriate geometry of the raw material, it increases the digestibility of the product. However, energy and logistical cost-effectiveness must be taken into account: for small batches in the home environment, the extended time and higher energy consumption may outweigh the qualitative benefits. Furthermore, the variety of results available in the literature to date (different temperatures, times, sample thicknesses, water addition in the package) provides a sensory or technological advantage over conventional methods, which may be misleading or statistically insignificant in some comparisons. This article synthesizes this knowledge and updates it with new findings.

In culinary practice, the sous vide method is used in restaurants, hotels, catering, and in the production of ready-to-eat food. It enables the preparation of dishes of consistent quality, retaining freshness, flavor, and nutritional value for an extended period. This process increases efficiency, minimizes raw material losses, and promotes sustainable food management by reducing food waste. Despite its greater time and energy consumption, SV can be considered a modern tool combining science, technology, and culinary art, redefining the standards of contemporary gastronomy, promoting health, quality, and innovation in meal preparation.

## Figures and Tables

**Table 1:** Conditions for preparing vegetables in the SV method.

Vegetables	Thickness or Portion	Temperature (°C)	Time (Min/Max)	Source
carrots, parsnips, potatoes, turnips, celery roots, beets	2.5–5 cm	84	2–5 h/4 h	[32]
asparagus, broccoli, corn, cauliflower, eggplant, onions, green beans, fennel, squash, fresh peas	up to 2.5 cm	84	30 min/1.5 h	
carrots	1 cm thick disks (cylinders), diameter range 2–4.5 cm	90	to core *P*_90_^10^ values > 4 min and <6 min	[37]
carrots root parsley	1 cm thick disks (cylinders)	80	10/20/30 min	[45]
		90		
carrots *	cylinders 1.5 cm in height × 2 cm in diameter	80 90	40/55/70 min 30/45/60 min	[28]
potatoes *	1.5 cm in height × 2 cm in diameter	80 90	25/30/35 min	
green beans	6–7 cm long pieces	80 90	40/50/60 min 20/30/40 min	
broccoli, green beans beetroots	200 g 200 g 300 g	85	15/30/45 min 30/60/90 min 45/90/190 min	[44]
pumpkin	3 cm cubes/500 g	90	30 min	[62]
borlotti beans, pearl barley, peas, red lentils	250 g/2 kg	65 74	10 h 4 h	[48]
*Brussels sprouts*	500 g	90	45–50 min	[55,56]

* Carrots and potatoes were blanched for 1 min at 100 °C before cooking to reduce the cooking time.

**Table 2:** Comparison of color changes in vegetables prepared using different culinary techniques.

Vegetables	Method	Color Change	Author
Vegetables rich in chlorophyll			
Broccoli	SV, B, B_<100°C_	SV broccoli is greener than that prepared in B.	Guillén et al. [39]
	SV, B, S, MV, SV_MV_, Gr	Broccoli prepared using the SV and Gr methods is less green than that prepared using the B, SV_MV_, and MV methods.	Martínez-Hernández et al. [51]
	SV	In the SV method, the color and consistency of broccoli deteriorated.	Czarnowska-Kujawska et al. [44]
	SV, S	No color differences between methods.	Lafarga et al. [52,53]
	SV	The most intense green color of broccoli occurs at high process parameters.	Pero et al. [54]
Kale	SV, S	The color of SV kale is darker than that prepared using the S method.	Lafarga et al. [52,53]
Asparagus	B, S, MV, SV_80_, SV_100_, SV_MV_	Asparagus cooked using the SV_MV_, MV, and SV_100_ methods is greener than that cooked using SV_80_, B, and S methods. The greatest color change is in the S and SV_80_ methods, and the least in MV.	Gonnella et al. [1]
Green beans and peas	CV, SV, B	In the SV method, the lightness L* and green color a* are less intense than in the CV method. The overall difference in color between the SV, B, and CV methods is not significant (*p* > 0.05)	Iborra-Bernad et al. [28,49]
	CV, SV, B	SV—better color protection than other methods.	Koç et al. [40]
Green peas	SV	In the SV method, the color and consistency deteriorate.	Czarnowska-Kujawska et al. [44]
*Brussels sprouts*	SV, S	The color of the SV *Brussels sprouts* is less green than the color of steamed ones. There are no differences in the lightness L* and yellowness b* parameters of the samples.	Rinaldi et al. [34]
Zucchini	SV, S, B	Zucchini prepared using the SV method is lighter, whiter, and has less brown peel.	Ilic et al. [31]
Pumpkin	SV, CV, B	CV has the least impact on the color. The CV and SV methods ensure the sensory properties of pumpkin cubes	Rinaldi et al. [63]
	SV, B, MV	The SV method resulted in a loss of intense pumpkin color, lower mean flavor and texture values, and lower overall acceptability compared to the B and MV methods.	Da Silva et al. [62]
Chicory	B, S, MV, SV	Chicory cooked using the SV method was lighter than the B, S, and MV samples. The SV chicory had a greener color than B and S, but less than MV. The color changed the least in the SV method and the most in the S method.	Renna et al. [68]
Vegetables rich in carotenoids			
Carrot	SV, CV, B	No color differences between cooking methods.	Rinaldi et al. [34]
	SV, S		Koç et al. [40]
	SV, B	SV carrots are lighter and redder than B carrots.	Araya et al. [41]
	SV, B	The highest values of the a* color parameter occur in carrots prepared using the SV method.	Stanikowski et al. [45]
	B, Scso, O, SV	Samples prepared in Scso have the highest L* value. Methods B and SV have the greatest influence on the value of the L* parameter. The SV and Scso methods have the lowest values of the a* parameter. The SV method decreases the C values—carrots were lighter and had a more grayish color. The smallest changes in the C value were obtained using the O method.	Narwojsz et al. [42]
	CV, SV, B	SV carrots change color less than those prepared using the B and CV methods.	Iborra-Bernad et al. [28]; Guillén et al. [39]
	B, SV	Carrots prepared using the SV method have a redder color than those prepared using the B method.	Patras et al. [43]
	B, S, SV	SV carrots were less yellow in color than carrots prepared using the S and B methods.	Kowalewicz et al. [33]
Vegetables rich in anthoxanthins			
Cauliflower	SV, S	No differences in color parameters L*, a*, and b*.	Lafarga et al. [52,53]
White cabbage			
Potatoes	B, S, SV	SV potatoes had the least color change.	Kowalewicz et al. [33]
Parsley		SV parsley was the lightness (L*) and had a higher b* parameter value than the one prepared in B.	
Vegetables rich in anthocyanins			
Red cabbage	SV, S	SV red cabbage is redder than that cooked in a steamer. The b* and L* color parameters did not differ.	Lafarga et al. [52,53]
	SV, B	Red cabbage SV is redder (contains twice as many anthocyanins) than prepared in B.	Iborra-Bernad et al. [67]
Beetroots	SV	Beetroot SV deterioration of color and consistency.	Czarnowska-Kujawska et al. [44]
Purple potatoes	SV, CV	In the SV method, the potatoes are redder than in the CV method. No differences in parameter b*.	Iborra-Bernad et al. [27]

SV_(x)_—sous-vide method in a water bath or convection-steam oven (x—process temperature, e.g., SV_80_, SV_100_); CV—cook-vide method; B—boiling in water in 100 °C; S—steaming; Scso—steaming in a convection steam oven, O—baking in oven, B_<100°C_—boiling in water at below 100 °C; MV—heat treatment in a microwave oven, SV_MV_—sous-vide method in the microwave; Gr—grilling.

**Table 3:** The effect of sous vide processing of selected vegetables on nutritional value, bioavailability, and digestibility.

Vegetables	Effect on Nutritional Value	Source
Broccoli, green beans, beetroots	Protection of mineral content	[44]
Pumpkin	In the SV method, the reduction in ascorbic acid and polyphenolic compounds and the reduction in carotenoid content occur to a greater extent than in a microwave oven.	[62]
Borlotti beans pearl barley peas, red lentils	The SV method allows for products with a higher content of metals (zinc, iron, potassium, copper) than those cooked using the conventional method, boiling in a pot.	[48]
Artichoke	The SV method affects the bioavailability and metabolism of polyphenols. Over 80% of the phenolic metabolites were excreted 4 h after their ingestion, and there was a high variability in the gut microbial metabolites formed in different individuals.	[64]
*Brussels sprouts*	SV was the most effective hydrothermal treatment for polyphenol retention and antioxidant activity, making it a better alternative to steaming. Using an in vitro model, a significant difference (*p* < 0.05) was demonstrated between the concentration of bioavailable polyphenolic compounds and the polyphenol content in the plant material before digestion.	[55]
	The SV method allows for a higher level of retention of individual compounds. Thanks to the use of lower process temperatures and vacuum packaging, SV cooking can be an alternative to conventional cooking, allowing for the retention of the higher nutritional value of *Brassica oleracea* var. *gemmifera* (content of dry matter, ash, crude fat, and most minerals at the level of the raw sample, *p* ≤ 0.05).	[56]
Green peas	A lower level of antioxidant activity loss was determined in the SV method. Similar losses of vitamin C were observed in the SV method and cook-vide, while losses of phenolic compounds were lower in the cook-vide than in the SV method.	[49]
Kale	Cooking using the SV resulted in higher losses of soluble substances and vitamin C, and lower losses of phenolic compounds compared to boiling.	[65]
Beetroot, red cabbage, red pepper, green pepper, and kale	The effect on polyphenol content in various vegetables depends on the temperature and time applied.	[66]
Chickory	SV cooking generally did not differ (*p* > 0.01) from boiling and steaming in terms of total phenol content	[68]

**Table 4:** The effect of the SV method on the quality of selected vegetables—summary.

Attribute	Carrots	Zucchini	Broccoli	Cauliflower	Potatoes	Green Beans	Spinach	Artichoke	Kale	Chicory
Processing yield	+	+	+	+	+	+	+	+	+	+/−
Water content	+	+	+	+	+	+	+	+	+	+
Vitamins	+	+	+	+	+/−	+	+	+	+	+
Minerals	+	+	+	+	+	+	+	+	+	+
Bioavailability	+	+	+	+	+	+	+	+	+	+
Digestion	+	+	+	+	+	+	+	+	+	+
Green color	−	+	+	NA	NA	+	+	+/−	+/−	+
Red and yellow colors	+	−	NA	+	+	NA	NA	NA	NA	NA
Taste and aroma	+	+	+	+	+	+	+/−	+	+	+
Texture	+	+/−	+/−	+/−	+	+	+/−	+	+/−	+
Overall quality/ acceptability	+	+	+	+	+	+	+	+	+	+
Microbiological quality	+	+	+	+	+	+	+	+	+	+
Extension of shelf life	+	+	+	+	+	+	+	+	+	+

+ positive, +/− neutral, − negative, NA—not applicable. Source: own study based on literature [14,28,29,33,39,40,42,44,45,46,52,53,55,56,62,68,88,91].

**Table 5:** SWOT analysis of the SV method.

Strengths	Weaknesses
- Nutritional Preservation—Vegetables prepared using the SV method lose less water-soluble vitamins (C, B) than those cooked conventionally. This is due to low temperature and a lack of contact with water. - More intense, natural taste and aroma due to vacuum packaging, and less water in the process. - Better texture (firmness, crispness) due to precise temperature control and protection of vegetables from overcooking. - A more attractive color due to the lack of direct contact with water, which favors the preservation of natural pigments (chlorophylls, carotenoids). - Repeatability and standardization of quality. - Less oxidation (e.g., of fats) and better microbiological safety. - Possibility of preparation in advance, cooled, and then heated to consumption temperature while maintaining quality.	- Longer preparation time than conventional methods. - It requires specialized equipment (circulator, vacuum sealer), which may be unaffordable for small businesses or households. - Risk of loss of vegetable texture (e.g., too soft) if incorrect process parameters are used. - No possibility of obtaining a crispy surface (no Maillard reaction). - Barriers to Consumer Acceptance. - Some consumers have concerns about: “boil in the bag”, processing, unnaturalness.” - Higher preparation costs (longer process time, specialized equipment required).
Opportunities	Threats
- Growing consumer interest in healthy eating. - Development of the market for ready-made, healthy RTE meals. - Vegetable SV products (purees, salad vegetables, plant-based dishes) are becoming more and more popular. - Possibility to introduce new premium products to the catering and retail sectors. - Perfect for box diets, catering, including mass catering: stable quality, low nutritional loss, high meal quality. - Reducing food losses (zero waste) allows the use of vegetables with a shorter shelf life, thanks to prior processing. - Possibility of personalizing the texture—in fine dining—the ability to set the “ideal degree of softness”.	- Low consumer awareness of SV cooking. - Concerns about the food safety of food prepared in bags (microplastics) at high temperatures. - The potential for microbial spoilage if not properly executed. - Competition between methods with a better image: steaming (considered “healthier”), grilling (considered “tastier”), baking in a convection oven. - High implementation and operating costs. - A dynamic market for plant-based technologies - New technologies (high-pressure, steam hybrid) can be considered more environmentally friendly.

Source: based on literature [14,28,29,33,39,40,42,44,45,46,52,53,54,55,56,62,84,88,91,95].

## Data Availability

The data is contained within the article. The data used to support the findings of this study can be made available by the corresponding author upon request.

## Abbreviations

The following abbreviations are used in this manuscript:

SV	sous-vide method
SV_(x)_	sous-vide method in a water bath or convection-steam oven (x—process temperature if more than one parameter was used)
SV_MV_	sous-vide method in the microwave
LTLT	low temperature, long time
HTST	high temperature, short time
B	boiling in water at 100 °C
B_<100°C_	boiling in water at below 100 °C
CV	Cook vide
S	steaming
Scso	steaming in a convection steam oven
MV	heat treatment in a microwave oven
O	baking in the oven
Gr	grilling
L*a*b*	CIE LAB color space parameters
ΔE	total color difference value
DPPH	2,2-Diphenyl-1-picrylhydrazyl
FRAP	Ferric Reducing Antioxidant Power
TPC	Total Phenolic Content
TP	Total Polyphenols
TAA	Total Antioxidant Activity
GAE	Gallic Acid Equivalents
ABTS	2,2′-azino-bis(3-ethylbenzothiazoline-6-sulfonic acid)
MUFA	Monounsaturated fatty acids
TVC	Total Viable Count
CFU	Colony Forming Units
GRA	glucoraphanin
GLS	glukosinolates
GIV	glucoraphenin
GNS	gluconasturtin
HPLC–UV–VIS	High-Performance Liquid Chromatography with UV-VIS detection
